# MicroRNA-379-5p plays a tumor-suppressive role in human bladder cancer growth and metastasis by directly targeting MDM2

**DOI:** 10.3892/or.2022.8413

**Published:** 2022-09-20

**Authors:** Deyao Wu, Xiaobing Niu, Jun Tao, Pengchao Li, Qiang Lu, Aiming Xu, Wei Chen, Zengjun Wang

Oncol Rep 57: 3502–3508, 2017; DOI: 10.3892/or.2017.5607

Following the publication of the above article, an interested reader drew to the authors' attention that the cell and invasion migration assay data featured in Figs. 2C and 5D contained two pairs of overlapping panels, such that the data appeared to have been derived from the same original sources, even though the data panels were intending to show the results from differently performed experiments. Moreover, there was also an instance of duplicated data panels comparing between the si-NC/cell invasion and si-NC/cell migration assay panels in Fig. 4C.

After having examined their original data, the authors have realized that inadvertent errors were made during the process of compiling these figures. Corrected versions of Figs. 2, 4 and 5, incorporating all the data from one of the repeated experiments, are shown opposite and on the next page. The authors all agree to the publication of this corrigendum, and are grateful to the Editor of *Oncology Reports* for allowing them the opportunity to publish this. They also regret any inconvenience caused to the readership of the Journal.

## Figures and Tables

**Figure 2.**
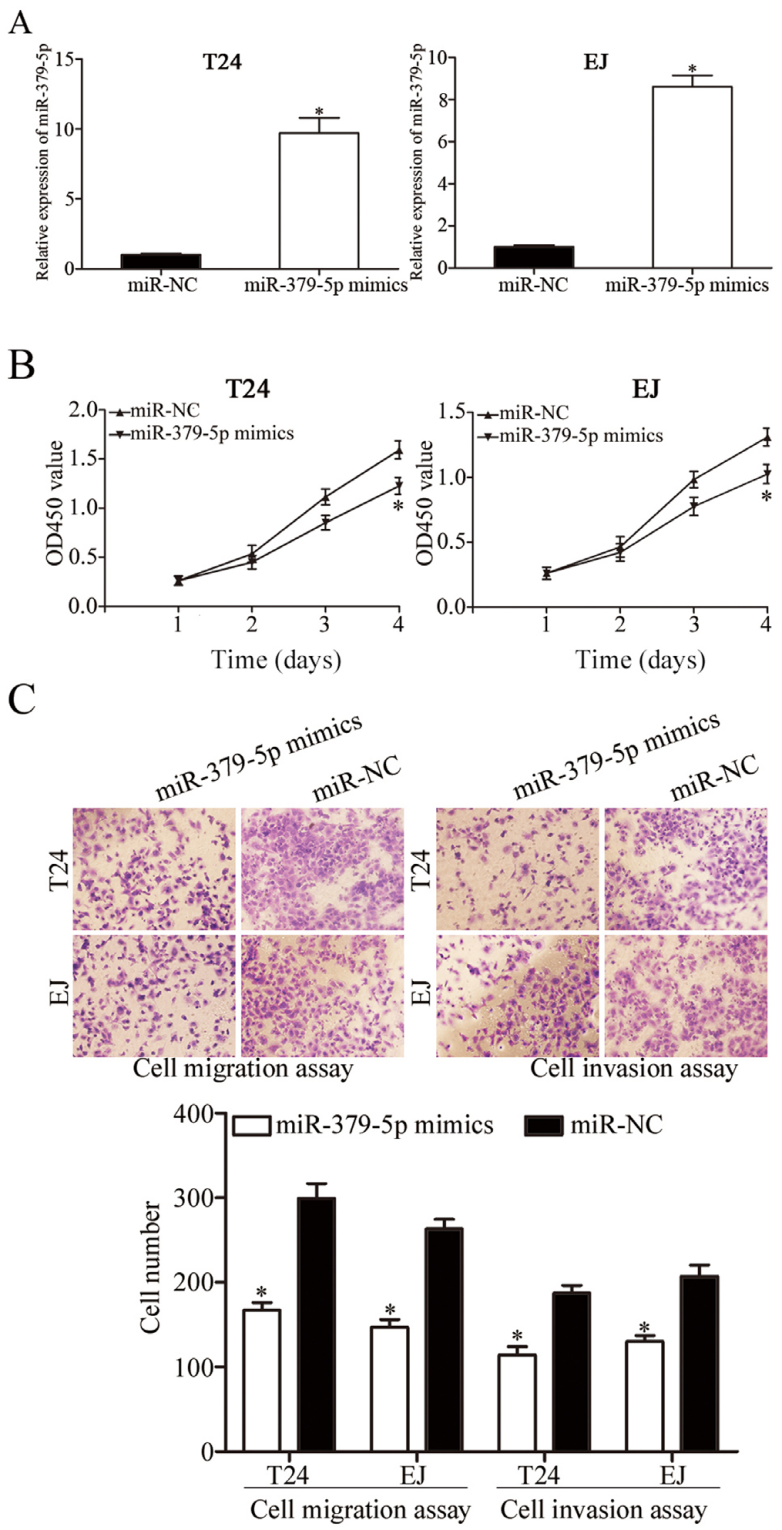
Restoration of miR-379-5p expression inhibits bladder cancer cell proliferation, migration and invasion. (A) RT-qPCR was conducted to assess transfection efficiency in T24 and EJ cells after transfection with miR-379-5p mimics or miR-NC. (B) CCK-8 assay was used to evaluate the proliferation of T24 and EJ cells after transfection with miR-379-5p mimics or miR-NC. (C) Resumption of the expression of miR-379-5p decreased the migration and invasion abilities of the T24 and EJ cells; ^*^P<0.05 compared with the control.

**Figure 4.**
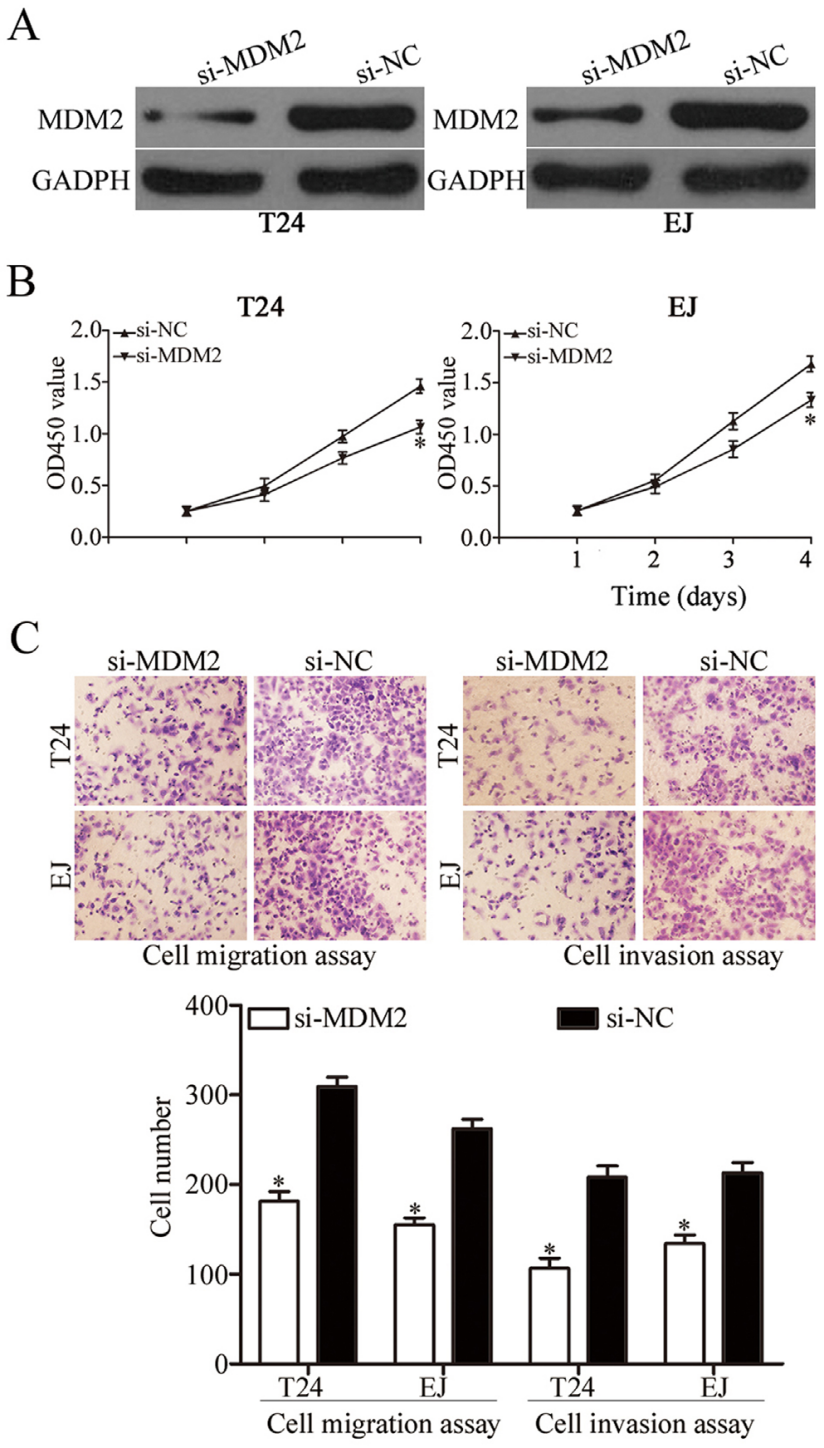
Effects of MDM2 knockdown on the biological behaviors of T24 and EJ cells. (A) Western blot analysis of MDM2 protein expression in the T24 and EJ cells transfected with si-MDM2 or si-NC. (B) CCK-8 assay showed that MDM2 knockdown suppressed T24 and EJ cell proliferation *in vitro*. (C) Downregulation of MDM2 reduced the migration and invasion abilities of T24 and EJ cells; ^*^P<0.05 compared with the control.

**Figure 5.**
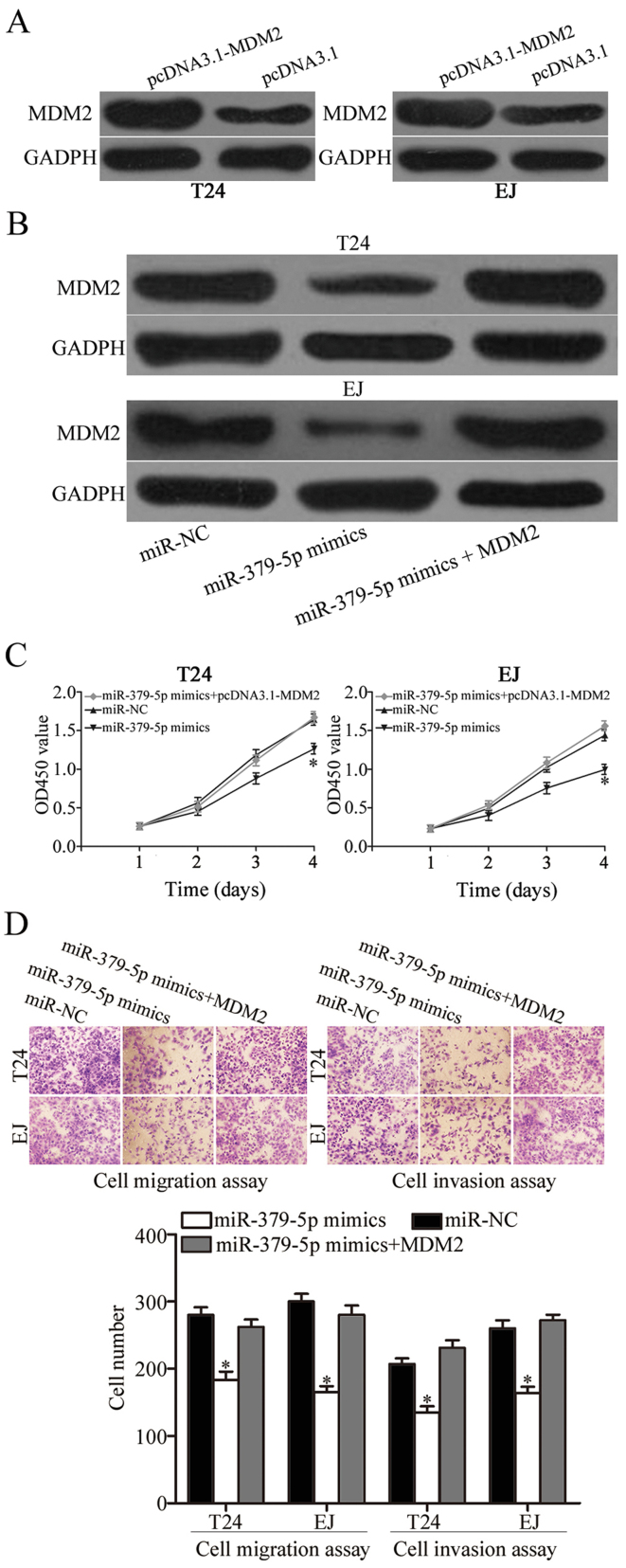
MDM2 is an important functional mediator of miR-379-5p in T24 and EJ cells. (A) Western blot results showed that MDM2 protein was upregulated in pcDNA3.1-MDM2-transfected T24 and EJ cells. (B) Western blot results showed that MDM2 protein in T24 and EJ cells was recovered after co-treatment with miR-379-5p mimics and pcDNA3.1-MDM2. (C) CCK-8 assay showed that the ectopic expression of MDM2 rescued the proliferation induced by miR-379-5p overexpression in T24 and EJ cells. (D) Cell migration and invasion assays revealed that the exogenous expression of MDM2 rescued the migration and invasion abilities induced by miR-379-5p overexpression in T24 and EJ cells; ^*^P<0.05 compared with the control.

